# Social trust and stress symptoms among older adults during the COVID-19 pandemic: evidence from Asia

**DOI:** 10.1186/s12877-022-02847-5

**Published:** 2022-04-15

**Authors:** Nan Jiang, Alfred M. Wu, Edmund W. Cheng

**Affiliations:** 1 Department of Social Work, Faculty of Arts and Social Sciences, National University of Singapore, Singapore, Singapore; 2 Lee Kuan Yew School of Public Policy, National University of Singapore, Singapore, Singapore; 3 Department of Public Policy, City University of Hong Kong, Kowloon Tong, Hong Kong

**Keywords:** Social trust, Stress, COVID-19, Cross-national survey, Asia

## Abstract

**Objectives:**

To investigate whether social trust is associated with more stress symptoms among middle-aged and older adults in six East and Southeast Asia regions during the COVID-19 pandemic.

**Methods:**

This multi-region study used cross-sectional survey data collected in May 2020. Participants were a probability-based internet sample of adults aged 55 or older.

**Results:**

Government trust was negatively associated with stress in Singapore and South Korea. Higher levels of health care trust were significantly associated with less stress in Singapore and Taiwan. Trust in neighbors was associated with a higher likelihood of stress in Hong Kong and a lower likelihood in Singapore. Social trust was not associated with stress in Japan or Thailand.

**Discussion:**

Findings suggest the level of social trust in relation to stress substantially varied by region. Interventions to strengthen trust during COVID-19 and other major health crises need to be tailored to fit regions’ unique circumstances.

**Supplementary Information:**

The online version contains supplementary material available at 10.1186/s12877-022-02847-5.

## Introduction

COVID-19 is a global health crisis with myriad implications for aging and the lives of older adults [13]. First, older adults have significantly higher infection rates and mortality risks than younger adults [24]. Moreover, adherence to physical and social distancing mandates may deplete older adults’ coping resources, putting them at high risk of social isolation and ultimately, increased stress [15]. Furthermore, older adults have experienced exacerbated ageism in the public discourse and institutional decision-making as a result of medical resource allocation [18]. Finally, inadequate or false information and intergenerational tension can further heighten stress [8, 32]. Combining these universal aspects with the added vulnerabilities faced by older adults, such as elevated health risks, mobility limitations, and economic hardship, the psychological impact of the COVID-19 pandemic could be substantial [19, 37].

Social trust is an important factor for explaining the substantial international differences in pandemic-related stress among older adults [3, 31]. As a critical component of social capital, social trust can be conceptualized as a “moral resource” that facilitates reciprocal exchanges in networks [16]. A growing body of research has linked social trust to mental health among older adults during the COVID-19 pandemic [9, 35], and mental health is often more strongly associated with trust than other dimensions of social capital [1, 34].

Despite the growing body of research, most studies on social trust and stress among older adults during the COVID-19 pandemic have been conducted in North Atlantic countries [9, 35]. The association between social trust and stress is understudied in East and Southeast Asia (ESA), although ESA has achieved notable success in controlling the pandemic [17]. Some factors that characterize social trust differences in North Atlantic countries may not be generalized to ESA, which is culturally collectivist and abides by strict norms [11]. Therefore, this study investigates the association between social trust and stress symptoms among older adults in six ESA regions during the COVID-19 pandemic.

### Literature review

#### COVID-19, stress, and social trust

The transactional model of stress and coping can be applied to understand the impact of COVID-19 as a stressor on older adults’ mental health [22, 37]. This theory posits that the stress experience occurrs in the interactions between a person and the broader context. According to this theory, the influence of the stressor depends on (a) how older adults appraise it as stressful and (b) how older adults cope with it, or the extent to which they can engage resources to combat the stress. If the coping resources are not sufficient and psychological arousal remains heightened, then maladaptive psychological outcomes are more likely to appear.

Burgeoning research has examined the detrimental consequences of the COVID-19 pandemic on the mental health of older adults [2, 8, 9, 15, 17, 35, 37]. Some studies suggested that age may protect against COVID-19 psychopathology because older adults have better coping skills due to life experience [5, 6, 18, 24, 27]. However, older adults are more vulnerable to the effects of the pandemic because of prior health conditions, decreased sensory awareness, physical impairments, and financial difficulties [4, 24]. Furthermore, some features of COVID-19 likely elevate its stressfulness. First, the lockdown measures and social distancing put older adults at risk of social isolation and loneliness, stressors that gain prevalence with age [15]. Moreover, the unpredictability of the timespan of the pandemic, who would be infected, as well as its long-term impacts, exacerbated feelings of uncertainty and uncontrollability [29]. In turn, this resulted in heightened stress responses among older adults, worrying about the effects of the pandemic, even if they were not themselves infected [36]. For these reasons, it is crucial to understand the consequences of the COVID-19 pandemic for the older population.

Social trust could be a coping resource for older adults. Social trust emanates from social capital and is a multidimensional concept [26]. Within society, we consider individuals’ trust in each other (interpersonal trust), as well as institutions and organizations (such as government and healthcare), referred to as institutional trust [21]. The two constituent components of social trust influence each other in a society [20, 30]. Potential mechanisms connecting social trust to stress include: (a) people with high levels of government trust are more likely to adhere to restrictions and support the required actions; (b) trust in the health care system enhances confidence in rapid epidemiological investigations and medical treatment; and (c) trust among neighbours buffers against stressors and increases faith in the community’s efficacy in containing the virus [14]. For older adults, high levels of social trust contribute to a sense of security and confidence that may ultimately reduce the effects of stressors. Social trust also benefits older people by providing social support, neighborhood cohesion, and other resources to buffer stress. Living in a community where others stand ready to help may make older adults feel less fearful [10]. Furthermore, high levels of social trust may facilitate better adherence and adaption to health-protective behaviors, such as the use of facemask and social distancing. Knowing that others act in the same way may in turn further reduce stress among older adults [36]. Empirical evidence therefore demonstrates that social trust is associated with lower stress among older adults [3, 17], and the lack of robust social trust during the COVID-19 pandemic was associated with more mental health problems [35].

#### COVID-19 and ESA

The ESA region has achieved notable success in controlling the COVID-19 pandemic, with effective implementation of non-pharmaceutical interventions to prevent the spread of the disease, such as facemask use, strict border controls, quarantine, social distancing, as well as widespread testing and contact tracing. This ultimately led to a comparatively low mortality rate [23]. In contrast to the North Atlantic region, older populations in ESA, especially those with chronic conditions, have been prioritized in the allocation of medical resources [3]. Another feature of ESA is its tight-knit culture, including strict norms and punishments for social deviance [12]. Moreover, because of its previous pandemic experience, ESA was prepared for COVID-19, with a high degree of coordination and strict adherence to social norms [11]. Finally, research from ESA has found little ageism, but high interest in intergenerational solidarity, in contrast to findings reported by studies conducted in the West [28, 39].

In general, the success of ESA stems from a combination of top-down approaches, with the government setting strong control policies, and bottom-up strategies, with the general public having strong social trust and therefore complies with government-directed mandates. However, research on the effects of social trust on late-life stress in ESA is lacking. It should not be assumed that relationships observed in North Atlantic countries are transferable to ESA settings. Our study sought to address these gaps by examining three indicators of social trust and their implications for stress among older adults across six ESA regions. Specifically, we hypothesized that older adults with a higher level of social trust in ESA would be less likely to experience stress during the pandemic.

## Methods

**Fig. 1:**
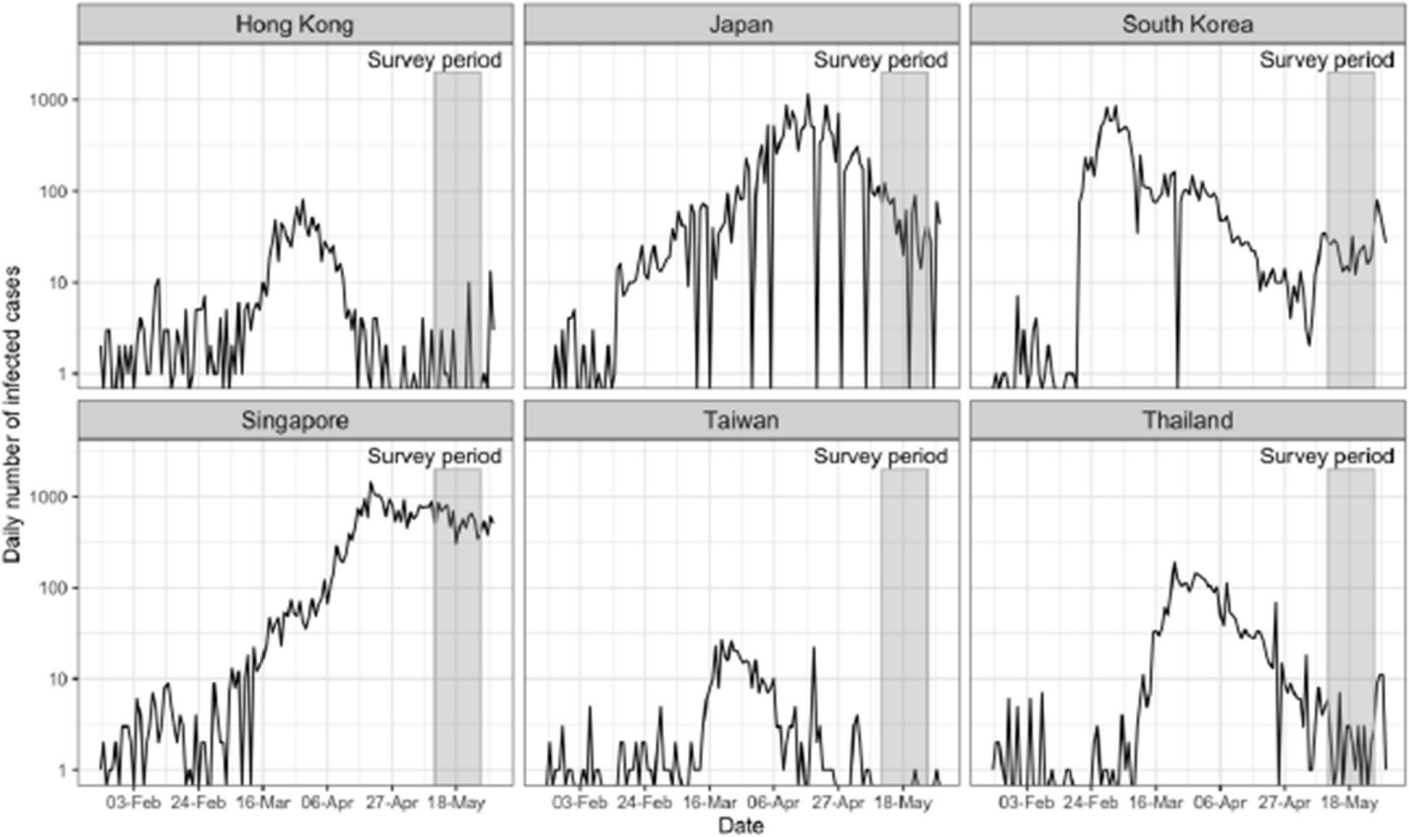
Daily Number of COVID-19 Cases and Survey Period, from February to May 2020

We conducted cross-national surveys to gauge public attitudes in six regions in ESA. The six regions are Hong Kong SAR, China; Japan; Singapore; South Korea; Taiwan, China (hereafter, Taiwan); and Thailand. Data collection occurred in May 2020, when these regions had been affected by COVID-19 after the outbreak in Wuhan and the World Health Organization (WHO) declared COVID-19 a global pandemic on March 11, 2020. As shown in Fig. 1, all six regions detected the first batch of COVID-19 cases in late January 2020. In late March, they were all exposed to the second wave of the outbreak as more imported cases from Europe and the United States were detected.

The survey adopted a probability-based internet panel [7], which has several advantages. First, the use of an internet panel is more feasible compared with face-to-face surveys during the pandemic. Second, it allows the standardization of measures to unpack the relationships between trust and stress under an almost laboratory environment. Third, it covers relatively under-studied regions that are exposed to comparable impacts of the pandemic, which helps to inform researchers and policymakers with timely evidence and provides an original data point for future research. We used a probability-based quota sampling strategy to ensure that the samples chosen match the population’s geographical and demographic characteristics released by the latest available census in each jurisdiction. The total representative sample included 12,062 participants, with approximately 2000 in each region [33]. We included individuals aged 55 years old or older (*N* = 2318) for this study, consistent with guidelines in major surveys [17].

### Measures

#### Stress symptoms

All participants in the study were asked questions from the Posttraumatic Stress Disorder Checklist. We chose five stress symptoms based on relevance to the COVID-19 pandemic. This scale has been reported to be highly consistent in general population samples [40]. Respondents rated their stress based on the following statements, with response options from 1 (*not at all*) to 7 (*very much*): “Since the COVID-19 outbreak, to what extent have you been (1) stressed about leaving home; (2) having repeated and disturbing thoughts or dreams about what is happening; (3) having difficulty concentrating; (4) having trouble falling or staying asleep, and (5) feeling irritable or having anger outburst?” The total score ranges from 5 to 35 (Cronbach’s α = .92).

#### Social trust measures

The survey asked the following questions: (1) “How much do you trust the following institutions to handle the coronavirus outbreak right? (a) government and (b) health care (including hospitals, doctors, and nurses)”; (2) “How much do you trust your neighbors have made an effort in containing the coronavirus outbreak?” Response options were on a Likert scale ranging from 1 (*totally do not trust*) to 7 (*totally trust*).

#### Covariates

Based on previous literature, we selected a set of key covariates available in the dataset based on their potential correlation to the study outcomes [9, 17, 24, 34, 35]. Demographics included age (55–59, 60–64, 65+), gender (male or female), education (secondary or below, tertiary, bachelor or above), residence status (urban or rural), employment status (yes or no), income (decile), living with spouse (yes or no), and living with children (yes or no). Participants were also asked whether they have chronic disease(s) (yes or no).

### Data analysis

The statistical analysis was conducted in Stata software (release 15.1; StataCorp, LP), while the descriptive analysis was conducted using unweighted numbers and proportions. Region-wise multivariable ordinary least squares (OLS) regression models adjusted for covariates were constructed to assess the association between social trust (exposure) and stress symptoms (outcomes). Separate OLS regression models were used for each exposure, rather than combining them in one regression, to avoid potentially multi-collinearity. We did not use multilevel models because such analyses can produce biased estimates when used with complex study designs.

In the second set of analyses, we modeled linear regressions within each region. The coefficient estimates for each region were combined into a random-effects meta-analysis to (1) visualize variability in the strength of associations, (2) examine the generalizability of findings across the constituent regions of ESA, and (3) confirm the robustness of findings using a second statistical approach. We calculated Higgins *I*^2^ statistics to evaluate the between-region heterogeneity levels that were not explained by sampling error. Cases with missing values (*n* = 10, 0.4%) were excluded in our complete analysis of the data. Results from OLS analyses are presented as coefficients with 95% confidence intervals. The level of statistical significance was set at *p* < 0.05 (two-sided). Based on our a priori power analysis, we calculated a 99% chance of detecting small effects (coefficient ≥ 0.6 for stress) in our 2-tailed OLS regression analyses (α = 0.05).

## Results

The sample characteristics of each region are shown in Table 1. The analytic sample consisted of 2308 individuals with 54.7% aged 55–59, 30.3% aged 60–64, and 15% aged 65 or older (48.6% were women and 51.4% were men). In total, 77.7% were urban residents, but this proportion was higher in South Korea (90.5%), Hong Kong SAR (89.3%), and Taiwan (87.9%). The average stress score ranged from 15.4 (Singapore) to South Korea (21.1). Singapore had the highest level of trust in government, health care, and neighbors (5.3, 6.0, and 5.8, respectively); Japan had the lowest government trust (3.6) and health care trust (5.3); and South Korea had the lowest neighbor trust (4.9). Bivariate comparisons revealed that participants with higher stress levels tended to be younger, have a bachelor’s degree or above, and live in urban areas.

**Table 1:** Descriptive statistics

	All		Hong Kong SAR		Japan		Singapore		South Korea		Taiwan		Thailand	
	Mean/%	SD	Mean/%	SD	Mean/%	SD	Mean/%	SD	Mean/%	SD	Mean/%	SD	Mean/%	SD
Stress (range:5–35)	17.4	0.2	19.7	0.5	16.4	0.3	15.4	0.4	21.1	0.4	15.7	0.4	16.4	0.5
Trust in government (range:1–7) ^*^	4.8	0.0	4.6	0.1	3.6	0.1	5.7	0.1	5.2	0.1	5.3	0.1	5.1	0.1
Trust in hospital (range:1–7) ^*^	5.7	0.0	5.8	0.1	5.3	0.0	6.0	0.1	5.9	0.1	5.8	0.1	6.1	0.1
Trust in neighbors (range:1–7) ^*^	5.4	0.0	5.3	0.1	5.4	0.0	5.8	0.1	4.9	0.1	5.1	0.1	5.8	0.1
Age^*^														
55–59	54.7		67.4		48.5		48.8		47.0		59.6		64.7	
60–64	30.3		24.7		28.2		33.7		40.2		29.7		25.6	
65+	15.0		7.9		23.3		17.6		12.7		10.7		9.7	
Gender														
Male	51.4		49.6		49.8		50.0		47.6		58.2		55.9	
Female	48.6		50.4		50.2		50.0		52.4		41.8		44.1	
Education^*^														
Secondary or below	27.5		46.6		30.5		36.4		5.6		19.5		18.9	
Tertiary	56.0		37.3		65.8		34.7		71.3		63.6		63.0	
Bachelor or above	16.5		16.2		3.7		29.0		23.1		16.9		18.1	
Residence status^*^														
Rural	22.3		10.7		44.2		17.1		9.5		12.1		27.3	
Urban	77.7		89.3		55.8		82.9		90.5		87.9		72.7	
Employment status														
Not employed	35.3		37.0		43.0		24.3		39.3		29.7		34.5	
Employed	64.7		63.0		57.0		75.7		60.7		70.3		65.5	
Income decile														
1	5.4		5.8		3.3		7.9		3.0		9.3		2.9	
2	6.8		2.2		6.5		13.4		5.6		6.5		5.5	
3	10.0		13.2		8.7		18.8		8.0		5.6		2.9	
4	10.0		9.6		9.3		15.1		9.8		10.2		3.8	
5	8.9		7.9		8.0		10.6		13.9		6.2		6.7	
6	9.3		7.9		9.2		11.4		10.7		8.2		7.6	
7	11.8		14.5		11.8		5.9		17.8		10.5		11.3	
8	9.4		9.3		8.0		3.0		13.3		14.7		10.5	
9	11.7		14.5		11.5		3.5		9.8		11.9		24.8	
10	8.5		9.9		6.3		3.0		5.9		10.5		21.8	
Income missing	8.2		5.2		17.3		7.4		2.4		6.5		2.1	
Living with spouse														
No	29.1		20.0		38.8		22.4		27.0		32.1		29.5	
Yes	70.8		80.0		61.2		77.6		73.0		67.9		70.5	
Living with children														
No	90.5		95.6		96.5		79.5		97.4		87.4		78.0	
Yes	9.5		4.4		3.5		20.5		2.6		12.6		22.0	
Have chronic disease(s)														
No	76.9		83.6		72.3		78.5		63.9		85.6		80.7	
Yes	23.1		16.4		27.7		21.5		36.1		14.4		19.3	
*N*	2308		366		597		404		344		356		241	

^*^ *p* < 0.05

The multivariable linear regression analyses adjusted for sociodemographic characteristics and health covariates showed that all of the three social trust components were negatively associated with stress (Table 2). The coefficient magnitude and significance of government trust, healthcare, and neighbors were strengthened (*β* = − 0.83, SE = .28, *P* < .01; *β* = − 1.55, SE = .37, *P* < .001; *β* = − 0.75, SE = .36, *P* < .05, respectively) when we interacted region by each measure of social trust.

**Table 2:** Trust and Stress Symptoms in Six Regions during COVID-19 Pandemic

	Trust in government		Trust in hospital		Trust in neighbors	
	M1	M2	M1	M2	M1	M2
Trust	−0.080	−0.825^**^	−0.298^*^	−1.551^***^	−0.078	−0.752^*^
	(0.101)	(0.275)	(0.149)	(0.370)	(0.140)	(0.360)
Region						
(ref. Singapore)						
Japan	1.653^**^	2.648	1.577^**^	7.016^**^	1.893^***^	1.799
	(0.581)	(1.796)	(0.555)	(2.687)	(0.551)	(2.646)
Hong Kong SAR	4.450^***^	3.849^*^	4.410^***^	10.563^***^	4.691^***^	8.571^**^
	(0.593)	(1.942)	(0.585)	(3.147)	(0.588)	(2.770)
South Korea	6.127^***^	5.854^**^	6.190^***^	0.791	6.332^***^	4.328
	(0.604)	(2.094)	(0.600)	(3.329)	(0.615)	(2.804)
Taiwan	0.519	2.502	0.517	1.409	0.611	2.726
	(0.588)	(2.193)	(0.586)	(3.267)	(0.598)	(2.722)
Thailand	1.625^*^	5.433^*^	1.693^*^	10.417^**^	1.651^*^	5.212
	(0.680)	(2.273)	(0.676)	(3.664)	(0.677)	(3.680)
Region*trust						
(ref. Singapore)						
Japan*trust		0.760^*^		1.466^**^		0.613
		(0.340)		(0.458)		(0.455)
Hong Kong SAR*trust		1.620^***^		2.548^***^		2.412^***^
		(0.349)		(0.526)		(0.482)
South Korea*trust		−0.015		1.166^*^		0.260
		(0.369)		(0.548)		(0.503)
Taiwan*trust		0.520		0.297		0.536
		(0.387)		(0.545)		(0.483)
Thailand*trust		1.299^**^		2.011^***^		1.176
		(0.405)		(0.595)		(0.618)
*N*		2308		2308		2308

Notes: Data are restricted to participants 55 years and older. Figures in each column are from a separate regression. The dependent variable is stress symptoms. Trust is the independent variable listed as column heading. All models are based on OLS regression and controlled for age, gender, education, resident status, employment status, income, perceived social support, whether have chronic diseases, whether live with spouse, and whether live with children. Robust standard errors in parentheses

^*^ *p* < 0.05, ^**^ *p* < 0.01, ^***^ *p* < 0.001

**Fig. 2:**
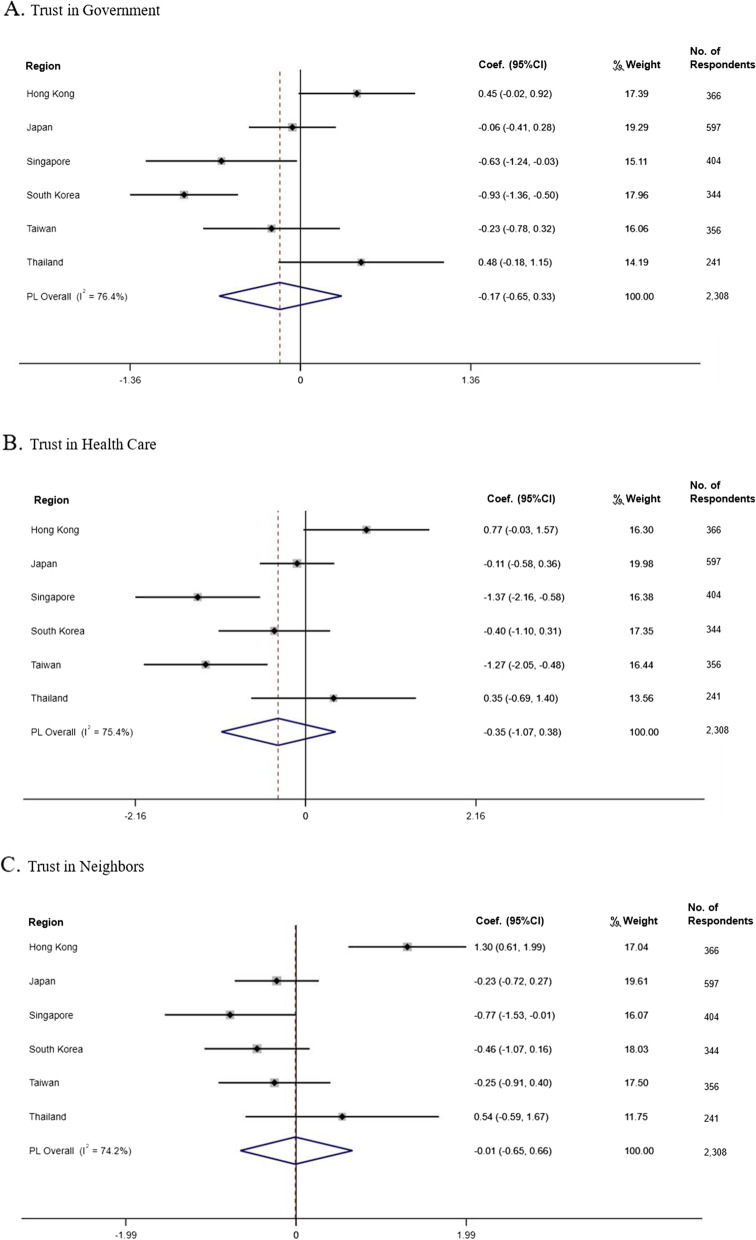
Region-wise Association Between Social Trust and COVID-19 Stress Symptoms. *Notes:* Associations were estimated with multivariable ordinary least squares regression, adjusting for age, gender, income level, education, employment status, residence status, presence of chronic diseases, whether live with spouse, and whether live with children. The overall estimates and weights were calculated by random-effects meta-analysis. Coef. refers to the regression coefficient; diamond, heterogeneity. 0 to 40%: might not be important; 40 to 75%: may represent moderate heterogeneity; 75 to 100%: considerable heterogeneity

Results from the linear regression analyses in each region and meta-analysis are shown in Fig. 2 (from A to C). We found a significant negative association between government trust and stress in Singapore (*b* = − 0.63, *SE* = 0.31, *p* < .05) and South Korea (*b* = − 0.93, *SE* = 0.22, *p* < .001; Fig. 2A). Figure 2B shows that higher levels of health care trust were only significantly associated with less stress in Singapore (*b* = − 1.36, *SE* = 0.40, *p* < .001) and Taiwan (*b* = − 1.26, *SE* = 0.40, *p* < .01). Furthermore, participants who had more trust in their neighbors had significantly higher likelihood levels of stress in Hong Kong (*b* = 1.299, *SE* = 0.35, *p* < .001), while significantly lower levels were observed in Singapore (*b* = − 0.77, *SE* = 0.39, *p* < .05; Fig. 2C). All three models showed high levels of heterogeneity among regions (*I*^2^ = 76, 95% confidence interval [CI] = 47, 89%; *I*^2^ = 76, 95% CI = 45, 89%; *I*^2^ = 75, 95% CI = 45, 89%, respectively). The associations between social trust components and stress was nonsignificant in Japan and Thailand (*p* > .05). Full model results are presented in Appendix Table A1-A3.

### Sensitivity analyses

In order to assess whether the region-wise association between social trust and stress was different by demographic or socioeconomic groups, we conducted additional interaction analyses in the fully adjusted model. Specifically, age enhanced the protective effect of social trust in Hong Kong and Japan. Moreover, social trust was positively associated with stress among older adults with higher education in Hong Kong and Taiwan. Government trust was associated with a greater risk of stress for high-income older adults in Japan. High socioeconomic status magnified the positive effect of social trust in Thailand, except for neighborhood trust. In addition, because perceived social support was considered the consequence of social trust [21], we did not include it in our main models. We reran the models using perceived social support as a covariate and found the substantive results remained the same. We also found a significant interaction between social trust and perceived social support in Japan, indicating a moderating effect. The tests of these sensitivity analyses are provided in Online Appendix Table A4-A8.

## Discussion

This was the first study to investigate the relationship between social trust and stress among older adults in ESA. Our major finding is that social trust was associated with stress in a large sample of adults aged 55 or older in ESA, but the level of social trust in relation to stress substantially varied by region. This is inconsistent with the research conducted to date in North Atlantic countries [17, 35]. Understanding what explains this variation is not only critical for the advancement of a theoretical framework but also beneficial to interventions aimed at addressing future pandemics.

Our empirical data shows regional variation in the strength of government trust associated with stress in later life. Compared with other regions in our analysis, Singapore and South Korea exhibited the highest levels of government trust and a significant negative association with stress [38]. Consistent with previous findings, stricter policies in the two regions may increase government trust among residents, which is critical for harnessing public cooperation, achieving the high rates of behavior adherence necessary for pandemic management, and in turn, buffering negative mental health consequences in later life [3, 23]. By contrast, Japan did not implement either stringent social distancing and mobility control policies or more comprehensive testing and contact tracing, especially at the early stage of the outbreak. Thus, it is not surprising that weaker policies and less individual compliance in Japan are associated with the lowest government trust among the five ESA regions and a nonsignificant link with stress.

We also found the magnitude of the health care trust–stress connection to be greater in Singapore and Taiwan, which have high average health care trust scores. Given that COVID-19 is highly contagious, sufficient capacity to treat patients and effective measures to limit case numbers may act as stress buffers, reinforcing the negative link between health care trust and stress. Both Singapore and Taiwan adopted policy strategies to drive down community transmission. However, infections in Taiwan were very low in 2020, with only 799 cases and 7 deaths. In Singapore, the number of local cases accelerated to 58,843, but death rate remained low at 30. This is in line with the previous finding that the stress exposure of older people is related aggregate counts of COVID-19 deaths rather than the percentage [17].

The present analyses demonstrate that a high level of neighbor trust mitigated stress among respondents during the current pandemic only in Singapore. Speculatively, social cohesiveness and a sense of community facilitate rapid and cooperative responses, which improve mental health. Furthermore, a lower level of stress may result from the public’s proper understanding of the scientific challenges and high compliance with non-pharmaceutical interventions in the community [14]. Surprisingly, our study found that neighbor trust was positively associated with stress in Hong Kong. One possibility is that trusting neighbors may be detrimental in societies that have political unrest and decreased social trust [1]. Older adults who blindly trust their neighbors in a context where everyone else is mistrusting may get taken advantage of. For example, these older adults may be more exposed to traumatic life events such as fraud, which increases the risk of stress. We have also confirmed in our sensitivity analyses that this finding may partly be explained by social inequality mechanisms, which warrant further exploration in future studies.

Finally, no significant associations between social trust and stress were found in Japan or Thailand. The large sample and limited adjustments in our analysis indicate that our null findings are not attributable to a lack of statistical power or over-specification. We would have the ability to detect small effects (power of .99 to detect a coefficient ≥ .60 for stress). The mechanisms underlying the nonsignificant association in Thailand and Japan are unclear. Speculatively, Thailand might have shown no association between social trust and stress because it is a less-developed region with less health care protection, among other institutional differences. In addition, protests against the government began in early 2020, which could be another potential cause. For Japan, our finding stands in contrast to previous evidence indicating a protective effect of social trust on mental health among older adults after natural disasters [25]. A potential explanation for our divergent findings could stem from our sample composition, which included individuals between 55 and 64 years old. Our sensitivity analyses indicated that adults aged 65 or older with high levels of social trust were less likely to perceive stress, which is consistent with prior research. We also found that social trust was significantly related to a lower level of stress for Japanese older adults who reported perceived social support, suggesting the enabling role of social support as posited by the transactional model of stress and coping [36, 37].

Although this pandemic has been unpredictable, prospective strengthening of social trust may reduce its detrimental effect on the mental health of older adults. The impact of this pandemic on stress depends on a combination of risk and resilience factors [37]. A high level of social trust is important in facilitating resilience and ameliorating the psychological arousal caused by this pandemic. However, political turmoil, ineffective policies, and weakening economic situations can erode social trust in private and public spheres, as people become less trusting. Social trust may lose its protective function as a buffer against stress. This study shows that even in circumstances that require social distancing, quarantine, and contact tracing, efforts to bolster social trust may be critical in both the immediate and long term. Therefore, maintaining social trust and building social capital to yield effective social cohesion in times of need represent resilient coping strategies for older adults, even when pandemics are not predictable.

This research suggests that interventions are needed to strengthen social trust. By their nature, pandemics create inconsistency and uncertainty of a temporal, spatial, and normative nature. Government responses should be sufficiently timely and effective in action. Older adults’ needs for economic, food, health care, and physical security should be prioritized and reliably met to create the conditions for social trust. In connection with consolidating social trust, enforcing strict rules, as well as emphasizing intergenerational solidarity and the existence of a broadly shared endeavor would be useful. Furthermore, a robust democratic infrastructure that allows community voices from older adults and pathways for these voices to be translated into decision-making can help promote trust. In addition, open access to relevant information expressed in age-friendly language can contribute to system transparency. Ultimately, community engagement can demonstrate that older adults are being heard and that their views and difficulties are being considered by policymakers [14].

This study has several limitations. First, our empirical data were limited, and the relationships were associational. Although we accounted for the correlational nature of the data, including adjusting for important covariates, causality cannot be inferred from the current analyses. Future research could address this issue of temporal ordering related to our focal associations. Second, our sample had a minor underrepresentation of younger age groups. To the extent that the perception of social trust and stress is different in these groups, our effect estimates may be slightly biased. Third, the data limitations prevented us from comprehensively examining the mechanisms that might explain the observed patterns in different regions. Potential confounders unavailable in these survey data may further explain the implications of social trust. Personality traits and coping styles may alter the effects of social trust through appraisal and stress reduction. In addition, it is possible that other unmeasured contextual factors, such as perceived neighborhood cohesion, may explain the substantial variation in the association between social trust and stress across these six regions.

## Conclusions

The present study contributes to the growing literature on social trust and mental health among older adults during the COVID-19 pandemic. Findings reinforce the importance of social trust as a support resource for mental health in later life. Further, the present study identifies various contextual settings in ESA that either amplify or reduce the protective effects of social trust on stress among older adults. As policymakers and researchers prepare to address the long-term effects of the COVID-19 pandemic around the world, the present study offers a roadmap for examining such effects in ESA. Interventions to strengthen trust during COVID-19 and future crises can be successful if they are tailored to fit the unique circumstances of various regions.

## Supplementary Information

**Additional file 1.** Appendix Table.

## Data Availability

All data generated or analyzed during this study may be obtained from Dr. Edmund Cheng.

## Abbreviations

ESA: East and Southeast Asia
OLS: Ordinary Least Squares
CI: Confidence Interval
