# Supplementary material for: Social trust and stress symptoms among older adults during the COVID-19 pandemic: evidence from Asia
Source: BMC Geriatr. 2022 Apr 15;22:330. doi: 10.1186/s12877-022-02847-5 (PMC9012251; doi:10.1186/s12877-022-02847-5)
Supplement: Supplementary file 1 — Additional file 1. Appendix Table. [file 12877_2022_2847_MOESM1_ESM.docx]

Appendix Table 1. Government Trust and COVID-19 Related Stress in Six Regions

|  | Hong Kong | Japan | Singapore | South Korea | Taiwan | Thailand |
| --- | --- | --- | --- | --- | --- | --- |
| Trust in government | 0.451 | -0.064 | -0.633^*^ | -0.931^***^ | -0.232 | 0.485 |
|  | (0.238) | (0.178) | (0.309) | (0.220) | (0.279) | (0.338) |
| Age group |  |  |  |  |  |  |
| (ref.55-59) |  |  |  |  |  |  |
| 60-64 | -1.960 | -2.248^***^ | -2.452^**^ | 1.109 | -1.393 | -1.371 |
|  | (1.042) | (0.672) | (0.942) | (0.788) | (0.935) | (1.381) |
| 65+ | -4.016^*^ | -2.172^**^ | -1.033 | -0.931 | -4.249^**^ | -1.214 |
|  | (1.614) | (0.759) | (1.176) | (1.184) | (1.430) | (1.948) |
| Female | 0.014 | 1.496^*^ | -1.471 | 2.193^**^ | -1.265 | 2.299^*^ |
|  | (0.895) | (0.599) | (0.863) | (0.795) | (0.858) | (1.134) |
| Educational attainment |  |  |  |  |  |  |
| (ref. secondary or below) |  |  |  |  |  |  |
| Tertiary | 3.376^***^ | -0.345 | -0.887 | 0.240 | -0.525 | -1.283 |
|  | (0.971) | (0.632) | (1.022) | (1.644) | (1.094) | (1.637) |
| Bachelor or above | 2.991^*^ | 4.440^**^ | -1.622 | 1.593 | -0.503 | -1.730 |
|  | (1.356) | (1.613) | (1.083) | (1.818) | (1.427) | (2.033) |
| Urban | 1.668 | 0.192 | -0.494 | 2.203 | -2.748^*^ | 1.096 |
|  | (1.411) | (0.581) | (1.101) | (1.268) | (1.342) | (1.286) |
| Employed | -3.021^**^ | 0.803 | 0.385 | 1.359 | 0.015 | 0.620 |
|  | (0.992) | (0.649) | (1.051) | (0.799) | (1.002) | (1.310) |
| Income decile |  |  |  |  |  |  |
| (ref. 1^st^) |  |  |  |  |  |  |
| 2 | -10.388^**^ | -0.311 | 1.311 | -2.240 | 3.932 | -3.168 |
|  | (3.425) | (1.860) | (1.927) | (2.589) | (2.071) | (4.209) |
| 3 | -3.280 | -0.748 | 1.557 | 0.009 | 4.217 | -7.054 |
|  | (2.218) | (1.786) | (1.826) | (2.484) | (2.199) | (4.676) |
| 4 | -2.295 | -0.582 | 0.012 | -0.125 | 1.908 | -2.359 |
|  | (2.352) | (1.776) | (1.904) | (2.427) | (1.872) | (4.465) |
| 5 | -6.445^**^ | 0.223 | 0.505 | -3.157 | 3.876 | -5.823 |
|  | (2.403) | (1.806) | (2.015) | (2.319) | (2.127) | (4.121) |
| 6 | -6.847^**^ | -0.672 | -0.230 | -1.219 | 3.209 | -1.741 |
|  | (2.387) | (1.767) | (1.975) | (2.442) | (1.985) | (4.070) |
| 7 | -5.968^**^ | -1.685 | 0.461 | -1.305 | 2.746 | -4.190 |
|  | (2.208) | (1.740) | (2.349) | (2.351) | (1.871) | (3.895) |
| 8 | -7.248^**^ | 0.967 | -4.797 | -2.422 | 1.699 | -3.219 |
|  | (2.317) | (1.834) | (2.874) | (2.374) | (1.752) | (3.931) |
| 9 | -7.722^***^ | -0.909 | 0.348 | -3.101 | 0.324 | -5.748 |
|  | (2.170) | (1.752) | (2.746) | (2.469) | (1.818) | (3.708) |
| 10 | -6.138^**^ | -2.161 | -1.541 | -1.823 | 2.980 | -5.637 |
|  | (2.285) | (1.897) | (2.838) | (2.652) | (1.918) | (3.750) |
| Income missing | -13.019^***^ | -0.597 | 0.021 | 1.947 | 4.411^*^ | -8.663 |
|  | (2.747) | (1.658) | (2.151) | (3.192) | (2.176) | (5.169) |
| Have at least one chronic disease | -2.670^*^ | 0.823 | 0.050 | 0.959 | -0.097 | 0.232 |
|  | (1.191) | (0.626) | (1.027) | (0.753) | (1.187) | (1.444) |
| Living with spouse | -1.971 | -0.202 | -1.488 | -0.696 | -0.278 | 0.601 |
|  | (1.052) | (0.673) | (1.081) | (0.993) | (0.925) | (1.261) |
| Living with children | -6.576^***^ | -0.883 | -1.586 | -3.469 | -3.510^**^ | -3.849^**^ |
|  | (1.077) | (1.521) | (2.020) | (2.560) | (1.234) | (1.363) |
| Constant | 23.373^***^ | 16.921^***^ | 21.240^***^ | 22.440^***^ | 18.744^***^ | 17.843^***^ |
|  | (2.975) | (1.765) | (2.643) | (2.914) | (2.245) | (4.116) |
| *N* | 366 | 597 | 404 | 344 | 356 | 241 |

Robust standard errors in parentheses.^*^ *p* < 0.05, ^**^ *p* < 0.01, ^***^ *p* < 0.001

Appendix Table 2. Healthcare System Trust and COVID-19 Related Stress in Six Regions

|  | Hong Kong | Japan | Singapore | South Korea | Taiwan | Thailand |
| --- | --- | --- | --- | --- | --- | --- |
| Trust in healthcare system | 0.769 | -0.109 | -1.367^***^ | -0.397 | -1.266^**^ | 0.353 |
|  | (0.407) | (0.240) | (0.403) | (0.361) | (0.400) | (0.532) |
| Age group |  |  |  |  |  |  |
| (ref.55-59) |  |  |  |  |  |  |
| 60-64 | -2.099^*^ | -2.240^***^ | -2.458^**^ | 1.058 | -1.212 | -1.389 |
|  | (1.045) | (0.673) | (0.929) | (0.804) | (0.919) | (1.388) |
| 65+ | -4.023^*^ | -2.089^**^ | -1.080 | -0.916 | -4.321^**^ | -1.138 |
|  | (1.618) | (0.761) | (1.163) | (1.198) | (1.399) | (1.955) |
| Female | -0.013 | 1.454^*^ | -1.564 | 1.568^*^ | -1.281 | 2.245^*^ |
|  | (0.898) | (0.600) | (0.842) | (0.791) | (0.837) | (1.137) |
| Educational attainment |  |  |  |  |  |  |
| (ref. secondary or below) |  |  |  |  |  |  |
| Tertiary | 3.520^***^ | -0.329 | -0.979 | 0.347 | -0.517 | -1.456 |
|  | (0.969) | (0.633) | (1.010) | (1.682) | (1.076) | (1.645) |
| Bachelor or above | 3.382^*^ | 4.690^**^ | -1.630 | 1.774 | -0.708 | -1.991 |
|  | (1.380) | (1.580) | (1.072) | (1.857) | (1.406) | (2.033) |
| Urban | 1.793 | 0.154 | -0.513 | 2.527 | -2.552 | 1.236 |
|  | (1.416) | (0.582) | (1.089) | (1.294) | (1.323) | (1.292) |
| Employed | -3.018^**^ | 0.778 | 0.250 | 1.265 | 0.096 | 0.529 |
|  | (0.998) | (0.650) | (1.041) | (0.815) | (0.980) | (1.314) |
| Income decile |  |  |  |  |  |  |
| (ref. 1^st^) |  |  |  |  |  |  |
| 2 | -10.118^**^ | -0.236 | 1.486 | -1.807 | 4.229^*^ | -3.078 |
|  | (3.640) | (1.866) | (1.907) | (2.660) | (2.040) | (4.224) |
| 3 | -2.506 | -0.681 | 1.624 | 0.272 | 4.234 | -6.819 |
|  | (2.289) | (1.793) | (1.806) | (2.544) | (2.155) | (4.699) |
| 4 | -1.917 | -0.541 | 0.155 | -0.509 | 1.554 | -1.936 |
|  | (2.406) | (1.781) | (1.881) | (2.492) | (1.844) | (4.469) |
| 5 | -5.934^*^ | 0.269 | 0.566 | -3.069 | 3.685 | -5.407 |
|  | (2.462) | (1.809) | (1.993) | (2.393) | (2.093) | (4.151) |
| 6 | -6.543^**^ | -0.573 | 0.103 | -0.899 | 3.314 | -1.540 |
|  | (2.440) | (1.780) | (1.955) | (2.510) | (1.946) | (4.091) |
| 7 | -5.192^*^ | -1.613 | 0.823 | -1.258 | 2.971 | -3.797 |
|  | (2.283) | (1.749) | (2.328) | (2.412) | (1.844) | (3.922) |
| 8 | -6.967^**^ | 1.034 | -4.391 | -2.340 | 1.620 | -3.120 |
|  | (2.371) | (1.841) | (2.849) | (2.428) | (1.718) | (3.945) |
| 9 | -7.257^**^ | -0.849 | 0.370 | -3.658 | 0.361 | -5.278 |
|  | (2.237) | (1.766) | (2.716) | (2.534) | (1.787) | (3.711) |
| 10 | -5.679^*^ | -2.129 | -1.061 | -1.988 | 2.707 | -5.283 |
|  | (2.351) | (1.900) | (2.813) | (2.698) | (1.886) | (3.758) |
| Income missing | -12.683^***^ | -0.407 | 0.259 | 1.907 | 4.173 | -8.222 |
|  | (2.792) | (1.668) | (2.129) | (3.280) | (2.145) | (5.195) |
| Have at least one chronic disease | -2.497^*^ | 0.863 | 0.158 | 0.984 | 0.078 | -0.145 |
|  | (1.199) | (0.626) | (1.005) | (0.768) | (1.169) | (1.425) |
| Living with spouse | -1.957 | -0.249 | -1.508 | -0.439 | -0.322 | 0.786 |
|  | (1.057) | (0.670) | (1.070) | (1.007) | (0.911) | (1.250) |
| Living with children | -6.516^***^ | -0.858 | -1.452 | -3.442 | -3.271^**^ | -3.928^**^ |
|  | (1.084) | (1.524) | (2.000) | (2.463) | (1.220) | (1.364) |
| Constant | 20.302^***^ | 17.199^***^ | 25.862^***^ | 19.991^***^ | 24.670^***^ | 18.041^***^ |
|  | (3.800) | (1.997) | (3.107) | (3.371) | (2.874) | (5.048) |
| *N* | 366 | 597 | 404 | 344 | 356 | 241 |

Robust standard errors in parentheses.^*^ *p* < 0.05, ^**^ *p* < 0.01, ^***^ *p* < 0.001

Appendix Table 3. Neighbor Trust and COVID-19 Related Stress in Six Regions

|  | Hong Kong | Japan | Singapore | South Korea | Taiwan | Thailand |
| --- | --- | --- | --- | --- | --- | --- |
| Trust in neighbors | 1.299^***^ | -0.226 | -0.770^*^ | -0.455 | -0.252 | 0.538 |
|  | (0.351) | (0.251) | (0.389) | (0.313) | (0.333) | (0.576) |
| Age group |  |  |  |  |  |  |
| (ref.55-59) |  |  |  |  |  |  |
| 60-64 | -2.351^*^ | -2.073^**^ | -2.516^**^ | 1.103 | -1.350 | -1.223 |
|  | (1.032) | (0.680) | (0.942) | (0.802) | (0.947) | (1.393) |
| 65+ | -3.682^*^ | -1.977^**^ | -1.075 | -0.909 | -4.061^**^ | -0.904 |
|  | (1.616) | (0.765) | (1.178) | (1.187) | (1.466) | (1.957) |
| Female | -0.008 | 1.563^**^ | -1.689^*^ | 1.449 | -1.315 | 2.228 |
|  | (0.887) | (0.604) | (0.850) | (0.784) | (0.860) | (1.142) |
| Educational attainment |  |  |  |  |  |  |
| (ref. secondary or below) |  |  |  |  |  |  |
| Tertiary | 3.411^***^ | -0.456 | -0.835 | 0.337 | -0.540 | -1.653 |
|  | (0.958) | (0.636) | (1.020) | (1.667) | (1.117) | (1.657) |
| Bachelor or above | 2.399 | 4.540^**^ | -1.664 | 1.757 | -0.495 | -2.175 |
|  | (1.344) | (1.601) | (1.084) | (1.842) | (1.451) | (2.038) |
| Urban | 1.855 | 0.373 | -0.574 | 2.553^*^ | -2.885^*^ | 1.437 |
|  | (1.372) | (0.589) | (1.101) | (1.283) | (1.373) | (1.301) |
| Employed | -2.353^*^ | 0.651 | 0.354 | 1.361 | 0.074 | 0.703 |
|  | (0.998) | (0.659) | (1.051) | (0.808) | (1.019) | (1.323) |
| Income decile |  |  |  |  |  |  |
| (ref. 1^st^) |  |  |  |  |  |  |
| 2 | -10.350^**^ | 0.098 | 1.365 | -1.395 | 3.877 | -3.859 |
|  | (3.382) | (1.856) | (1.926) | (2.662) | (2.112) | (4.294) |
| 3 | -3.911 | -0.485 | 1.478 | 0.300 | 4.418 | -7.594 |
|  | (2.224) | (1.792) | (1.823) | (2.518) | (2.253) | (4.724) |
| 4 | -3.023 | -0.354 | 0.168 | -0.449 | 1.887 | -2.423 |
|  | (2.335) | (1.778) | (1.902) | (2.466) | (1.881) | (4.501) |
| 5 | -7.700^**^ | 0.356 | 0.899 | -3.129 | 3.931 | -5.997 |
|  | (2.386) | (1.811) | (2.017) | (2.361) | (2.143) | (4.142) |
| 6 | -7.718^**^ | -0.289 | -0.080 | -0.739 | 3.127 | -2.281 |
|  | (2.373) | (1.782) | (1.974) | (2.495) | (1.989) | (4.115) |
| 7 | -6.204^**^ | -1.250 | 0.493 | -0.900 | 2.844 | -4.426 |
|  | (2.212) | (1.749) | (2.348) | (2.393) | (1.889) | (3.922) |
| 8 | -7.966^***^ | 0.948 | -4.826 | -2.275 | 1.706 | -4.000 |
|  | (2.311) | (1.849) | (2.872) | (2.408) | (1.763) | (3.994) |
| 9 | -8.583^***^ | -0.667 | 0.510 | -3.646 | 0.451 | -5.902 |
|  | (2.170) | (1.768) | (2.743) | (2.506) | (1.841) | (3.744) |
| 10 | -5.740^*^ | -2.598 | -2.039 | -1.988 | 2.998 | -5.921 |
|  | (2.299) | (1.901) | (2.829) | (2.668) | (1.930) | (3.793) |
| Income missing | -12.694^***^ | -0.480 | 0.593 | 1.851 | 4.418^*^ | -9.146 |
|  | (2.702) | (1.657) | (2.170) | (3.242) | (2.231) | (5.217) |
| Have at least one chronic disease | -2.339 | 0.826 | 0.399 | 0.931 | -0.170 | -0.013 |
|  | (1.190) | (0.630) | (1.013) | (0.762) | (1.218) | (1.427) |
| Living with spouse | -2.003 | -0.110 | -1.288 | -0.333 | -0.385 | 0.882 |
|  | (1.057) | (0.673) | (1.086) | (1.004) | (0.939) | (1.246) |
| Living with children | -6.261^***^ | -0.931 | -1.733 | -3.004 | -3.517^**^ | -3.849^**^ |
|  | (1.067) | (1.551) | (2.020) | (2.455) | (1.254) | (1.379) |
| Constant | 18.734^***^ | 17.672^***^ | 21.730^***^ | 19.808^***^ | 18.871^***^ | 17.468^***^ |
|  | (3.309) | (2.006) | (3.002) | (3.076) | (2.374) | (4.699) |
| *N* | 366 | 597 | 404 | 344 | 356 | 241 |

Robust standard errors in parentheses.^*^ *p* < 0.05, ^**^ *p* < 0.01, ^***^ *p* < 0.001

Appendix Table 4. Associations between Social Trust Indicators and Stress by Region. Interaction with Age Groups (*N*=2,308)

|  | Singapore | Japan | Hong Kong | South Korea | Taiwan | Thailand |
| --- | --- | --- | --- | --- | --- | --- |
| *Panel 1. Government trust* | |  |  |  |  |  |
| Trust | -0.860^**^ | -0.047 | 0.877 | -1.136^***^ | -0.226 | -0.058 |
|  | (0.250) | (0.242) | (0.492) | (0.301) | (0.363) | (0.416) |
| Age |  |  |  |  |  |  |
| (ref. 55-59) |  |  |  |  |  |  |
| 60-64 | -2.552 | -2.341 | 4.179 | -1.797 | -2.363 | -9.004^*^ |
|  | (3.880) | (1.680) | (2.751) | (2.548) | (3.385) | (4.186) |
| 65+ | -8.174 | -1.700 | 0.357 | -1.016 | -1.187 | -9.498 |
|  | (4.912) | (1.922) | (3.987) | (3.607) | (5.136) | (6.318) |
| trust*age |  |  |  |  |  |  |
| (ref. 55-59) |  |  |  |  |  |  |
| 60-64 | 0.018 | 0.026 | -1.340^*^ | 0.562 | 0.182 | 1.517 |
|  | (0.665) | (0.421) | (0.558) | (0.467) | (0.615) | (0.781) |
| 65+ | 1.259 | -0.128 | -0.990 | 0.013 | -0.552 | 1.561 |
|  | (0.841) | (0.481) | (0.865) | (0.663) | (0.900) | (1.127) |
|  |  |  |  |  |  |  |
| *Panel 2: Hospital trust* | |  |  |  |  |  |
| Trust | -1.840^**^ | -0.072 | 0.848 | -0.403 | -0.924 | 0.296 |
|  | (0.606) | (0.307) | (0.492) | (0.501) | (0.522) | (0.648) |
| Age |  |  |  |  |  |  |
| (ref. 55-59) |  |  |  |  |  |  |
| 60-64 | -3.693 | -0.966 | -4.367 | 0.984 | 2.240 | -5.288 |
|  | (5.563) | (3.175) | (6.324) | (4.891) | (5.426) | (7.305) |
| 65+ | -11.791 | -2.729 | 2.806 | -0.985 | 3.531 | 14.254 |
|  | (6.422) | (3.691) | (7.115) | (6.322) | (7.222) | (17.316) |
| trust*age |  |  |  |  |  |  |
| (ref. 55-59) |  |  |  |  |  |  |
| 60-64 | 0.210 | -0.242 | 0.381 | 0.012 | -0.593 | 0.637 |
|  | (0.913) | (0.591) | (1.070) | (0.811) | (0.912) | (1.188) |
| 65+ | 1.792 | 0.117 | -1.236 | 0.012 | -1.351 | -2.442 |
|  | (1.056) | (0.676) | (1.252) | (1.040) | (1.219) | (2.732) |
|  |  |  |  |  |  |  |
| *Panel 3: Neighbor trust* | |  |  |  |  |  |
| Trust | -0.343 | -0.631 | 1.344^**^ | -0.479 | -0.377 | 0.613 |
|  | (0.560) | (0.348) | (0.416) | (0.440) | (0.410) | (0.731) |
| Age |  |  |  |  |  |  |
| (ref. 55-59) |  |  |  |  |  |  |
| 60-64 | 3.490 | -3.675 | -3.273 | 0.208 | -7.283 | 1.584 |
|  | (5.407) | (3.157) | (5.206) | (3.365) | (4.033) | (7.755) |
| 65+ | -0.605 | -11.139^**^ | -0.263 | 1.380 | 5.840 | -4.045 |
|  | (6.474) | (3.757) | (5.907) | (5.489) | (6.295) | (11.162) |
| trust*age |  |  |  |  |  |  |
| (ref. 55-59) |  |  |  |  |  |  |
| 60-64 | -1.018 | -0.301 | 0.159 | 0.184 | 1.143 | -0.482 |
|  | (0.904) | (0.571) | (0.930) | (0.666) | (0.761) | (1.312) |
| 65+ | -0.083 | -1.655^*^ | -0.710 | -0.454 | -1.822 | 0.535 |
|  | (1.083) | (0.665) | (1.171) | (1.068) | (1.145) | (1.870) |
|  |  |  |  |  |  |  |
| *N* | 366 | 597 | 404 | 344 | 356 | 241 |

*Notes*: Data are restricted to participants 55 years and older. Figures in each column are from a separate regression. The dependent variable is stress symptoms. Trust is the independent variable listed as panel heading. All models are based on OLS regression and controlled for age, gender, education, residence status, employment status, income, whether live with spouse, whether live with children, and whether have chronic diseases. Robust standard errors in parentheses.^*^ *p* < 0.05, ^**^ *p* < 0.01, ^***^ *p* < 0.001.

Appendix Table 5. Associations between Social Trust Indicators and Stress by Region. Interaction with Perceived Social Support (*N*=2,308)

|  | Singapore | Japan | Hong Kong | South Korea | Taiwan | Thailand |
| --- | --- | --- | --- | --- | --- | --- |
| *Panel 1. Government trust* | |  |  |  |  |  |
| Trust | -0.652 | -0.995^***^ | -0.008 | -1.572^**^ | 0.002 | 0.181 |
|  | (0.404) | (0.290) | (0.571) | (0.496) | (0.462) | (0.631) |
| Support | -5.541 | -1.815 | 3.527 | -0.488 | -8.852^**^ | 3.003 |
|  | (3.213) | (1.437) | (2.901) | (2.889) | (3.040) | (3.999) |
| trust*support | 0.124 | -1.446^***^ | 0.473 | 0.751 | -0.394 | 0.482 |
|  | (0.552) | (0.359) | (0.627) | (0.544) | (0.558) | (0.733) |
|  |  |  |  |  |  |  |
| *Panel 2: Hospital trust* | |  |  |  |  |  |
| Trust | -1.289^*^ | -0.177 | 0.822 | -0.278 | -0.338 | 0.328 |
|  | (0.515) | (0.420) | (0.820) | (0.988) | (0.723) | (1.019) |
| Support | -6.019 | 2.950 | 6.527 | 4.552 | -13.074^*^ | 4.966 |
|  | (4.524) | (2.726) | (5.404) | (6.245) | (5.085) | (7.268) |
| trust*support | 0.022 | 0.088 | -0.161 | -0.229 | -1.107 | 0.079 |
|  | (0.746) | (0.509) | (0.937) | (1.055) | (0.854) | (1.173) |
|  |  |  |  |  |  |  |
| *Panel 3: Neighbor trust* | |  |  |  |  |  |
| Trust | -0.517 | -0.482 | 0.284 | -1.460^*^ | -0.549 | -0.411 |
|  | (0.505) | (0.397) | (0.714) | (0.704) | (0.561) | (0.856) |
| Support | -9.549^*^ | 2.161 | -0.561 | -3.043 | -6.924^*^ | -0.333 |
|  | (4.332) | (2.770) | (4.208) | (3.974) | (3.418) | (6.654) |
| trust*support | -0.565 | 0.237 | 1.137 | 1.222 | 0.013 | 0.988 |
|  | (0.728) | (0.502) | (0.805) | (0.787) | (0.673) | (1.134) |
|  |  |  |  |  |  |  |
| *N* | 366 | 597 | 404 | 344 | 356 | 241 |

*Notes*: Data are restricted to participants 55 years and older. Figures in each column are from a separate regression. The dependent variable is stress symptoms. Trust is the independent variable listed as panel heading. All models are based on OLS regression and controlled for age, gender, education, residence status, employment status, income, whether live with spouse, whether live with children, and whether have chronic diseases. Robust standard errors in parentheses.^*^ *p* < 0.05, ^**^ *p* < 0.01, ^***^ *p* < 0.001.

Appendix Table 6. Associations between Social Trust Indicators and Stress by Region. Interaction with Residence Status (*N*=2,308)

|  | Singapore | Japan | Hong Kong | South Korea | Taiwan | Thailand |
| --- | --- | --- | --- | --- | --- | --- |
| *Panel 1. Government trust* | |  |  |  |  |  |
| Trust | -0.985 | 0.126 | 0.579 | 0.202 | 0.211 | 0.564 |
|  | (0.711) | (0.257) | (0.702) | (0.833) | (0.741) | (0.532) |
| Urban | -3.022 | 1.521 | 2.260 | 9.047 | -0.183 | 1.079 |
|  | (4.495) | (1.390) | (3.641) | (4.889) | (4.090) | (3.726) |
| trust*urban | 0.474 | -0.325 | -0.219 | -1.236 | -0.546 | 0.474 |
|  | (0.777) | (0.349) | (0.749) | (0.859) | (0.791) | (0.777) |
|  |  |  |  |  |  |  |
| *Panel 2: Hospital trust* | |  |  |  |  |  |
| Trust | -0.178 | -0.423 | -0.174 | 0.294 | -0.649 | 0.826 |
|  | (0.857) | (0.355) | (1.457) | (1.064) | (0.988) | (0.851) |
| Urban | 7.703 | -2.602 | -4.127 | 7.842 | 0.566 | 5.294 |
|  | (5.758) | (2.581) | (8.972) | (6.973) | (6.153) | (6.763) |
| trust*urban | -1.355 | 0.547 | 0.945 | -0.870 | -0.578 | -0.692 |
|  | (0.950) | (0.476) | (1.520) | (1.131) | (1.073) | (1.073) |
|  |  |  |  |  |  |  |
| *Panel 3: Neighbor trust* | |  |  |  |  |  |
| Trust | -0.612 | -0.445 | -0.340 | -2.213^*^ | 0.213 | 1.810 |
|  | (0.901) | (0.353) | (1.140) | (0.938) | (0.889) | (1.116) |
| Urban | 0.787 | -0.675 | -7.461 | -7.155 | 1.261 | 14.079 |
|  | (5.911) | (2.675) | (6.559) | (5.124) | (4.851) | (7.663) |
| trust*urban | -0.212 | 0.209 | 1.671 | 1.940 | -0.853 | -2.172 |
|  | (0.989) | (0.483) | (1.203) | (0.989) | (0.944) | (1.266) |
|  |  |  |  |  |  |  |
| *N* | 366 | 597 | 404 | 344 | 356 | 241 |

*Notes*: Data are restricted to participants 55 years and older. Figures in each column are from a separate regression. The dependent variable is stress symptoms. Trust is the independent variable listed as panel heading. All models are based on OLS regression and controlled for age, gender, education, residence status, employment status, income, whether live with spouse, whether live with children, and whether have chronic diseases. Robust standard errors in parentheses.^*^ *p* < 0.05, ^**^ *p* < 0.01, ^***^ *p* < 0.001.

Appendix Table 7. Associations between Social Trust Indicators and Stress by Region. Interaction with Education (*N*=2,308)

|  | Singapore | Japan | Hong Kong | South Korea | Taiwan | Thailand |
| --- | --- | --- | --- | --- | --- | --- |
| *Panel 1. Government trust* | |  |  |  |  |  |
| Trust | -0.339 | -0.137 | 0.190 | -2.137 | -0.545 | 2.004^**^ |
|  | (0.495) | (0.313) | (0.314) | (1.337) | (0.552) | (0.768) |
| Education |  |  |  |  |  |  |
| (ref. secondary or below) |  |  |  |  |  |  |
| Tertiary | 4.480 | -0.747 | 3.549 | -6.160 | -2.354 | 9.353 |
|  | (3.854) | (1.506) | (2.418) | (7.588) | (3.548) | (4.787) |
| Bachelor or above | -3.930 | 1.632 | -6.442 | -4.863 | 0.293 | 2.741 |
|  | (4.270) | (3.746) | (3.768) | (7.749) | (4.792) | (5.456) |
| trust*education |  |  |  |  |  |  |
| (ref. secondary or below) |  |  |  |  |  |  |
| Tertiary | -0.899 | 0.092 | -0.092 | 1.217 | 0.429 | -2.135^*^ |
|  | (0.661) | (0.382) | (0.485) | (1.366) | (0.639) | (0.879) |
| Bachelor or above | 0.422 | 0.634 | 1.997^**^ | 1.209 | -0.013 | -0.853 |
|  | (0.721) | (0.921) | (0.742) | (1.393) | (0.892) | (1.014) |
| *Panel 2: Hospital trust* | |  |  |  |  |  |
| Trust | -0.228 | 0.322 | 0.336 | -1.724 | -1.357 | 1.548 |
|  | (0.618) | (0.411) | (0.592) | (1.894) | (0.852) | (0.961) |
| Education |  |  |  |  |  |  |
| (ref. secondary or below) |  |  |  |  |  |  |
| Tertiary | 8.651 | 3.772 | 2.834 | -7.983 | 0.020 | 10.505 |
|  | (5.148) | (2.728) | (5.439) | (11.620) | (5.717) | (7.775) |
| Bachelor or above | 9.965 | -4.328 | -4.845 | -3.820 | -10.784 | 4.223 |
|  | (6.269) | (6.891) | (5.826) | (12.018) | (8.046) | (7.950) |
| trust*education |  |  |  |  |  |  |
| (ref. secondary or below) |  |  |  |  |  |  |
| Tertiary | -1.546 | -0.787 | 0.050 | 1.435 | -0.010 | -2.045 |
|  | (0.844) | (0.502) | (0.916) | (1.933) | (0.957) | (1.258) |
| Bachelor or above | -1.889 | 1.614 | 1.505 | 0.961 | 1.932 | -1.078 |
|  | (1.020) | (1.255) | (1.022) | (2.001) | (1.391) | (1.299) |
| *Panel 3: Neighbor trust* | |  |  |  |  |  |
| Trust | -0.771 | -0.527 | 1.447^**^ | 0.888 | -1.824^*^ | 1.877 |
|  | (0.571) | (0.443) | (0.513) | (1.519) | (0.737) | (1.138) |
| Education |  |  |  |  |  |  |
| (ref. secondary or below) |  |  |  |  |  |  |
| Tertiary | -6.404 | -0.855 | 5.848 | 6.893 | -5.751 | 8.462 |
|  | (5.230) | (2.978) | (4.107) | (7.897) | (4.246) | (7.850) |
| Bachelor or above | 4.624 | -11.559 | 4.751 | 11.345 | -11.803^*^ | 15.371 |
|  | (5.233) | (6.378) | (4.925) | (8.522) | (4.893) | (9.520) |
| trust*education |  |  |  |  |  |  |
| (ref. secondary or below) |  |  |  |  |  |  |
| Tertiary | 1.007 | 0.059 | -0.533 | -1.275 | 1.187 | -1.872 |
|  | (0.880) | (0.538) | (0.750) | (1.554) | (0.828) | (1.353) |
| Bachelor or above | -1.042 | 3.007^*^ | -0.406 | -1.877 | 2.485^*^ | -3.103 |
|  | (0.879) | (1.175) | (0.885) | (1.660) | (0.964) | (1.634) |
| *N* | 366 | 597 | 404 | 344 | 356 | 241 |

*Notes*: Data are restricted to participants 55 years and older. Figures in each column are from a separate regression. The dependent variable is stress symptoms. Trust is the independent variable listed as panel heading. All models are based on OLS regression and controlled for age, gender, education, residence status, employment status, income, whether live with spouse, whether live with children, and whether have chronic diseases. Robust standard errors in parentheses.^*^ *p* < 0.05, ^**^ *p* < 0.01, ^***^ *p* < 0.001.

Appendix Table 8. Associations between Social Trust Indicators and Stress by Region. Interaction with Income (*N*=2,308)

|  | Singapore | Japan | Hong Kong | South Korea | Taiwan | Thailand |
| --- | --- | --- | --- | --- | --- | --- |
| *Panel 1. Government trust* | |  |  |  |  |  |
| Trust | -2.479^*^ | -2.405^*^ | 2.199 | -1.098 | 0.342 | -2.566 |
|  | (1.115) | (0.975) | (1.273) | (1.548) | (0.763) | (1.814) |
| Income decile |  |  |  |  |  |  |
| (ref. 1st) |  |  |  |  |  |  |
| 2 | -15.066 | -8.580 | 11.088 | -7.938 | 5.246 | -29.206^*^ |
|  | (7.747) | (4.462) | (10.004) | (8.803) | (6.308) | (11.322) |
| 3 | -11.741 | -8.450 | 3.349 | -2.921 | -1.239 | -11.480 |
|  | (7.792) | (4.440) | (7.992) | (8.631) | (7.279) | (12.781) |
| 4 | -16.034 | -11.617^**^ | 2.403 | -1.592 | 4.561 | -35.048^*^ |
|  | (8.223) | (4.304) | (7.819) | (8.901) | (6.168) | (13.748) |
| 5 | -3.179 | -11.703^**^ | 2.246 | -0.641 | 5.121 | -12.554 |
|  | (7.947) | (4.381) | (7.954) | (8.366) | (7.565) | (12.425) |
| 6 | -12.291 | -8.116 | 6.385 | -5.462 | 20.910^**^ | -28.660^*^ |
|  | (7.910) | (4.261) | (8.230) | (8.316) | (7.142) | (11.184) |
| 7 | -14.520 | -12.699^**^ | 3.437 | -2.940 | 0.130 | -19.428 |
|  | (9.321) | (4.215) | (7.848) | (8.160) | (5.311) | (10.222) |
| 8 | -35.328^*^ | -9.933^*^ | 8.591 | -1.809 | 5.866 | -17.942 |
|  | (17.826) | (4.423) | (7.901) | (8.539) | (5.067) | (10.390) |
| 9 | -10.268 | -9.623^*^ | 4.413 | -5.148 | 4.517 | -18.968 |
|  | (8.676) | (4.244) | (7.793) | (8.945) | (5.729) | (10.322) |
| 10 | -24.894 | -9.496^*^ | 4.723 | 16.216 | 8.239 | -27.153^**^ |
|  | (24.074) | (4.522) | (7.980) | (10.407) | (6.438) | (10.224) |
| Income missing | -9.639 | -9.969^*^ | -4.506 | 9.606 | 7.330 | -59.929^*^ |
|  | (9.869) | (4.069) | (8.718) | (10.460) | (6.737) | (26.728) |
| trust*income decile |  |  |  |  |  |  |
| (ref. 1st) |  |  |  |  |  |  |
| 2 | 2.752^*^ | 2.388^*^ | -4.673^*^ | 1.190 | -0.248 | 4.785^*^ |
|  | (1.319) | (1.188) | (2.276) | (1.747) | (1.171) | (2.161) |
| 3 | 2.049 | 1.985 | -1.138 | 0.540 | 0.952 | 0.680 |
|  | (1.308) | (1.175) | (1.420) | (1.710) | (1.314) | (2.503) |
| 4 | 2.732 | 2.885^*^ | -0.675 | 0.317 | -0.728 | 5.588^*^ |
|  | (1.414) | (1.126) | (1.431) | (1.711) | (1.183) | (2.479) |
| 5 | 0.521 | 3.009^**^ | -1.428 | -0.420 | -0.458 | 1.007 |
|  | (1.369) | (1.123) | (1.478) | (1.650) | (1.416) | (2.414) |
| 6 | 1.928 | 1.786 | -2.456 | 0.841 | -3.276^*^ | 5.276^*^ |
|  | (1.354) | (1.114) | (1.529) | (1.648) | (1.274) | (2.151) |
| 7 | 2.564 | 2.767^*^ | -1.693 | 0.360 | 0.349 | 2.727 |
|  | (1.569) | (1.096) | (1.414) | (1.613) | (1.021) | (1.966) |
| 8 | 5.270 | 2.705^*^ | -3.177^*^ | -0.126 | -0.974 | 2.422 |
|  | (2.962) | (1.142) | (1.448) | (1.686) | (0.972) | (2.002) |
| 9 | 1.667 | 2.129 | -2.279 | 0.402 | -0.862 | 2.438 |
|  | (1.504) | (1.100) | (1.400) | (1.718) | (1.081) | (1.967) |
| 10 | 3.909 | 1.985 | -1.988 | -3.304 | -1.257 | 3.965^*^ |
|  | (3.854) | (1.158) | (1.457) | (1.976) | (1.211) | (1.952) |
| Income missing | 1.600 | 2.475^*^ | -1.207 | -1.435 | -0.818 | 9.677 |
|  | (1.679) | (1.076) | (1.639) | (1.984) | (1.299) | (5.097) |
| *Panel 2: Hospital trust* | |  |  |  |  |  |
| Trust | -2.667^*^ | -0.582 | 2.275 | -1.367 | -0.257 | 14.581^*^ |
|  | (1.141) | (1.056) | (1.298) | (1.869) | (1.185) | (6.979) |
| Income decile |  |  |  |  |  |  |
| (ref. 1st) |  |  |  |  |  |  |
| 2 | -11.000 | -4.424 | 25.637 | -23.429 | -3.351 | 76.820 |
|  | (9.186) | (6.952) | (19.667) | (13.298) | (12.799) | (48.647) |
| 3 | -9.140 | -4.165 | -6.588 | -7.776 | -3.872 | 116.049^*^ |
|  | (8.717) | (7.042) | (9.791) | (13.577) | (12.426) | (49.731) |
| 4 | -16.590 | -9.250 | 0.565 | 1.287 | 6.847 | 64.993 |
|  | (9.064) | (7.519) | (12.113) | (12.860) | (9.001) | (58.888) |
| 5 | 2.945 | -8.765 | 1.952 | -12.494 | 4.268 | 94.574^*^ |
|  | (8.994) | (6.601) | (11.583) | (12.649) | (12.689) | (45.111) |
| 6 | -14.586 | -8.812 | -0.899 | -11.455 | 27.494^*^ | 75.697 |
|  | (10.085) | (7.140) | (10.417) | (12.665) | (11.172) | (45.862) |
| 7 | 1.945 | -2.092 | 14.070 | -5.583 | 15.263 | 79.683 |
|  | (14.522) | (6.531) | (10.027) | (11.269) | (11.028) | (44.599) |
| 8 | -54.227^**^ | -3.751 | 3.912 | -6.426 | 5.482 | 94.145^*^ |
|  | (20.099) | (6.445) | (12.585) | (11.095) | (8.263) | (46.906) |
| 9 | -3.000 | 5.251 | 10.023 | -0.127 | 4.181 | 93.172^*^ |
|  | (11.404) | (6.456) | (10.313) | (13.566) | (9.251) | (44.865) |
| 10 | -21.852 | -10.302 | 10.769 | 47.004^*^ | 5.023 | 76.093 |
|  | (22.968) | (6.715) | (10.368) | (19.549) | (10.555) | (45.075) |
| Income missing | -10.336 | -1.639 | 14.502 | -21.020 | 14.294 | 59.441 |
|  | (13.070) | (5.972) | (13.007) | (20.379) | (10.506) | (60.958) |
| trust*income decile |  |  |  |  |  |  |
| (ref. 1st) |  |  |  |  |  |  |
| 2 | 1.985 | 0.838 | -6.268 | 3.660 | 1.243 | -13.055 |
|  | (1.518) | (1.366) | (3.604) | (2.312) | (2.119) | (7.637) |
| 3 | 1.572 | 0.532 | 0.801 | 1.339 | 1.361 | -19.981^*^ |
|  | (1.430) | (1.373) | (1.616) | (2.354) | (2.094) | (7.957) |
| 4 | 2.734 | 1.520 | -0.422 | -0.172 | -1.076 | -11.261 |
|  | (1.501) | (1.483) | (2.036) | (2.238) | (1.577) | (9.076) |
| 5 | -0.475 | 1.541 | -1.196 | 1.658 | -0.244 | -16.389^*^ |
|  | (1.499) | (1.310) | (1.985) | (2.198) | (2.198) | (7.127) |
| 6 | 2.307 | 1.310 | -0.840 | 1.808 | -4.135^*^ | -12.291 |
|  | (1.653) | (1.371) | (1.709) | (2.223) | (1.870) | (7.252) |
| 7 | -0.159 | -0.126 | -3.284 | 0.818 | -2.204 | -13.396 |
|  | (2.350) | (1.284) | (1.682) | (2.021) | (1.857) | (7.031) |
| 8 | 8.063 | 0.659 | -1.794 | 0.716 | -0.766 | -15.775^*^ |
|  | (4.172) | (1.271) | (2.091) | (1.994) | (1.426) | (7.376) |
| 9 | 0.387 | -1.348 | -2.883 | -0.484 | -0.686 | -15.814^*^ |
|  | (1.932) | (1.265) | (1.710) | (2.345) | (1.583) | (7.071) |
| 10 | 3.344 | 1.494 | -2.764 | -7.983^*^ | -0.582 | -13.110 |
|  | (3.541) | (1.331) | (1.735) | (3.295) | (1.850) | (7.099) |
| Income missing | 1.704 | 0.125 | -4.309^*^ | 3.753 | -1.956 | -10.640 |
|  | (2.128) | (1.188) | (2.142) | (3.358) | (1.826) | (10.195) |
|  |  |  |  |  |  |  |
| *Panel 3: Neighbor trust* | |  |  |  |  |  |
| Trust | -2.575 | -0.642 | 2.980^*^ | -2.092 | -1.904^*^ | -8.426^*^ |
|  | (1.520) | (0.854) | (1.304) | (2.329) | (0.932) | (4.086) |
| Income decile |  |  |  |  |  |  |
| (ref. 1st) |  |  |  |  |  |  |
| 2 | -8.467 | 4.535 | 20.737 | -0.856 | -14.621 | -89.505^**^ |
|  | (10.142) | (6.290) | (11.352) | (13.641) | (8.965) | (28.723) |
| 3 | -8.532 | -2.507 | -8.729 | -7.222 | -12.297 | -46.924 |
|  | (9.995) | (7.002) | (8.592) | (11.704) | (7.998) | (40.310) |
| 4 | -20.517 | -3.804 | 5.143 | -7.475 | -5.420 | -49.897 |
|  | (10.736) | (6.183) | (9.094) | (11.242) | (6.463) | (28.586) |
| 5 | -5.353 | -7.002 | 10.385 | -9.954 | -8.443 | -48.966^*^ |
|  | (12.842) | (6.445) | (9.909) | (11.104) | (8.939) | (23.551) |
| 6 | -11.397 | -3.219 | -1.133 | -19.016 | 1.850 | -50.827^*^ |
|  | (10.860) | (5.792) | (9.311) | (11.687) | (7.034) | (23.659) |
| 7 | -16.271 | -12.193 | 6.298 | -3.140 | -6.868 | -51.669^*^ |
|  | (13.529) | (6.515) | (8.549) | (10.780) | (6.839) | (22.260) |
| 8 | -36.850^*^ | -1.550 | 8.479 | -7.036 | -2.932 | -64.358^**^ |
|  | (17.156) | (6.351) | (9.721) | (10.890) | (5.719) | (23.880) |
| 9 | -5.561 | 4.607 | 1.018 | -9.924 | -5.888 | -47.344^*^ |
|  | (11.760) | (6.241) | (8.502) | (12.315) | (6.834) | (21.820) |
| 10 | -3.322 | -9.716 | 5.743 | -17.399 | -5.987 | -42.654 |
|  | (17.172) | (6.454) | (9.373) | (11.729) | (6.298) | (22.126) |
| Income missing | -7.477 | -2.149 | 13.777 | -17.008 | 0.823 | -55.767 |
|  | (14.165) | (5.459) | (9.448) | (15.007) | (9.539) | (32.557) |
| trust*income decile |  |  |  |  |  |  |
| (ref. 1st) |  |  |  |  |  |  |
| 2 | 1.585 | -0.766 | -6.219^**^ | 0.315 | 3.813^*^ | 15.115^**^ |
|  | (1.734) | (1.193) | (2.297) | (2.799) | (1.739) | (5.162) |
| 3 | 1.511 | 0.266 | 0.842 | 1.662 | 3.380^*^ | 7.958 |
|  | (1.698) | (1.334) | (1.559) | (2.582) | (1.538) | (7.052) |
| 4 | 3.458 | 0.498 | -1.604 | 1.652 | 1.399 | 8.896 |
|  | (1.817) | (1.194) | (1.674) | (2.503) | (1.321) | (5.223) |
| 5 | 1.099 | 1.170 | -3.242 | 1.637 | 2.387 | 8.264 |
|  | (2.150) | (1.205) | (1.793) | (2.483) | (1.737) | (4.557) |
| 6 | 1.802 | 0.346 | -1.149 | 3.732 | 0.286 | 9.368^*^ |
|  | (1.857) | (1.107) | (1.695) | (2.560) | (1.391) | (4.530) |
| 7 | 2.881 | 1.784 | -2.347 | 0.649 | 1.901 | 9.134^*^ |
|  | (2.305) | (1.219) | (1.577) | (2.425) | (1.345) | (4.327) |
| 8 | 5.542 | 0.235 | -3.046 | 1.111 | 0.912 | 11.206^*^ |
|  | (2.858) | (1.202) | (1.779) | (2.454) | (1.164) | (4.554) |
| 9 | 0.898 | -1.099 | -1.759 | 1.477 | 1.345 | 8.198 |
|  | (2.015) | (1.156) | (1.536) | (2.688) | (1.345) | (4.250) |
| 10 | 0.215 | 1.267 | -2.148 | 3.276 | 1.682 | 7.367 |
|  | (3.005) | (1.223) | (1.740) | (2.577) | (1.256) | (4.295) |
| Income missing | 1.334 | 0.210 | -4.984^**^ | 4.016 | 0.675 | 8.939 |
|  | (2.355) | (1.052) | (1.786) | (3.215) | (1.832) | (5.827) |
|  |  |  |  |  |  |  |
| *N* | 366 | 597 | 404 | 344 | 356 | 241 |

*Notes*: Data are restricted to participants 55 years and older. Figures in each column are from a separate regression. The dependent variable is stress symptoms. Trust is the independent variable listed as panel heading. All models are based on OLS regression and controlled for age, gender, education, residence status, employment status, income, whether live with spouse, whether live with children, and whether have chronic diseases. Robust standard errors in parentheses.^*^ *p* < 0.05, ^**^ *p* < 0.01, ^***^ *p* < 0.001.
